# Insights from a measles outbreak root cause analysis in Ethiopia - 2024

**DOI:** 10.11604/pamj.supp.2025.51.1.47804

**Published:** 2025-06-13

**Authors:** Mikias Alayu Alemu, Sisay Temesgen Dema, Habtamu Alemay Anteneh, Habtamu Tilahun Guade, Abay Hagos Gebrekidan, Amare Mengistu Mersha, Gebre Asmamaw Lakew, Yonas Asefa Tufa, Endale Zemene Shibeshi, Gizaw Teka Nibzane, Fasil Teshager Hailemariam, Yamlak Gindola Kita, Fentaw Yimam, Wondimu Bekele Ayana, Tirhas Hailekiros, Kefyalew Amene, Rahel Atakilti, Emebet Alemu, Ommer Yeshaw, Sarai Malumo, Melkamu Ayalew Kokebe, Mesfin Wossen Getaneh, Melkamu Abte Afele, Balcha Girma Masresha

**Affiliations:** 1 Ethiopian Public Health Institute (EPHI), Addis Ababa, Ethiopia,; 2 World Health Organization, Country Office for Ethiopia, Addis Ababa, Ethiopia,; 3 African Field Epidemiology Network (AFENET), Addis Ababa, Ethiopia,; 4 Oromia Regional Health Bureau, Addis Ababa, Ethiopia,; 5 Amhara Regional Health Bureau, Bahir Dar, Ethiopia,; 6 Ministry of Health-Ethiopia, Addis Ababa, Ethiopia,; 7 Vaccine-preventable Diseases Program, World Health Organization, Regional Office for Africa, Brazzaville, Congo

**Keywords:** Measles, vaccination, outbreak, Ethiopia, root cause analysis

## Abstract

**Introduction:**

Ethiopia has been implementing measles elimination strategies since 2003, including periodic supplemental immunisation activities every 2-3 years, and the introduction of a second dose measles vaccine in 2018. At national level, the coverage for first and second dose measles vaccine is 61% and 53% respectively. In the past decade, populations displacements and service disruptions due to natural and man-made disasters, exacerbated by the impact of the COVID-19 pandemic on the sub-optimally functioning immunisation system, have contributed to multiple measles outbreaks in the country.

**Methods:**

between July and August of 2024, a total of 44 woredas conducted a measles outbreak response vaccination campaign. Among these, 11 woredas were selected purposively to implement an outbreak root cause analysis exercise. Analysis of data and document reviews were done at woreda and health facility levels, along with interview of immunization program officers at different administrative levels, and interviews of care givers of confirmed measles cases.

**Results:**

in 2024, a total of 4664 confirmed measles cases and 24 deaths were reported to the national level from the 11 woredas. Despite reported high administrative vaccine coverage at the woreda level, only 56% of the measles cases had received at least one dose of measles vaccine, as seen during household visits. More than half (55%) of the 11 woredas do not have any facility that offers daily vaccination services, due to staff shortage (36%), concerns of vaccine wastage (36%), and lack of operational funding (18%). Twelve (44%) out of the 27 health posts and one-third (8 of 24) of the health centers do not give measles vaccines in every vaccination session. Cancellations of vaccination sessions were reported from 24 health centers and 27 health posts in 4 woredas due to various reasons including staff shortage, vaccine stockout, lack of means of transportation for field trips and electric power interruption. Non vaccination due to a combination of these multiple factors was identified as the root cause of the measles outbreaks.

**Conclusion:**

there are cross-cutting systemic factors that contributed to the measles outbreaks in various woredas, alongside contextual factors which differ from one health facility to another and between woredas. It is important that these findings are used to develop tailored efforts to strengthen routine immunisation services within the service catchment areas. Root cause analysis should be implemented as part of every outbreak investigation and response efforts.

## Introduction

The Federal Republic of Ethiopia is administratively organized into 12 regional states and 2 city administrations. These are further divided into more than 1260 woredas (the equivalent of districts), made up of more than 32000 kebeles (the equivalent of villages) - the lowest administrative unit in the country [1]. The United Nations Population Division (UNPD) estimates the country’s total population for 2024 to be 132,059,767 with an estimated 4,016,093 births in the year. The infant mortality rate was estimated to be 31.9 per 1000 live births while the under-five child mortality rate was 43.2 per 1000 [2]. The country has a three-tier healthcare delivery system. As of December 2021, there are 353 public hospitals, 3,706 public health centers and 17,561 health posts in the country [3]. On average, one woreda has two to three health centers and a primary hospital. Each health center is linked to 5 satellite health posts, which provide services to a population of approximately 25,000 [1]. Each woreda health office and each health center/hospital is expected to have one dedicated Expanded Program on Immunization (EPI) program officer, and one officer responsible for Public Health Emergency Management (PHEM).

According to the Ethiopia Service Provision Assessment (2021-2022), about 75% of health facilities in Ethiopia offer child immunization services, and Pentavalent, polio, measles, and BCG vaccines were available in more than 85% of the facilities offering child vaccination services [4]. The first dose of measles vaccine (MCV1) is given at 9 months of age, while the second measles dose (MCV2) is provided at 15 months starting in 2019. The country currently uses 10-dose vials for measles vaccination. Measles-rubella (MR) vaccine is not yet introduced in the national vaccination schedule as of December 2024 [1].

According to the WHO UNICEF national immunization coverage estimates (WUENIC) for 2023, Ethiopia has MCV1 and MCV2 coverage levels of 61% and 53% respectively [5]. The most recent available Ethiopia Mini-DHS report from 2019 indicated that MCV1 coverage was 58.5% at national level, ranging from 29.6% in Afar Region to 90.6% in Addis Ababa. The DHS also demonstrated coverage differences between urban (MCV1 coverage of 78.1%) and rural populations (MCV1 coverage of 50%) [6]. Ethiopia has been implementing measles elimination strategies since its first nationwide preventive measles mass vaccination campaign in 2003, which was followed by periodic follow up supplemental immunisation activities every 2 - 3 years [7]. In the past decade, different parts of Ethiopia have repeatedly experienced population displacement and disruption of health care services as a result of climate change, natural disasters and internal as well as cross-border conflicts. In 2025, it is estimated that 28.6 million people require humanitarian assistance [8]. These displacements and service disruptions, exacerbated by the impact of the COVID-19 pandemic on the sub-optimally functioning immunisation system, have contributed to multiple measles outbreaks in the country. In 2023 and 2024, Ethiopia reported the incidence of confirmed measles to be 154 and 276 cases per million population respectively [9].

The Ethiopian national measles surveillance guideline defines a confirmed measles outbreak as the occurrence of three or more laboratory-confirmed suspected measles cases in one month per woredas (equivalent to district) or 100,000 population in a geographic area or a health facility catchment area [10,11]. The World Health Organization (WHO) recommends that, as part of measles outbreak response, countries should implement root cause analysis and take corrective programmatic action [12]. Root cause analysis (RCA) is a systematic process for identifying the underlying causes of problems, enabling organizations to implement effective, long-term solutions by focusing on the root causes rather than just addressing symptoms. This problem-solving method uses various techniques to drill down and get to the source of problems. One of the most utilized techniques involves repeatedly asking “why” in order to go beneath the surface level explanations and arrive at the root causes [13]. Following one of the measles outbreak response vaccination campaigns which took place from July to August 2024 with the support of the Measles and Rubella Partnership (M&RP), a measles outbreak root cause analysis exercise was conducted in the woredas targeted by the response campaign. This manuscript summarizes the findings of the RCA.

## Methods

Between July and August of 2024, a total of 44 woredas conducted measles outbreak response vaccination campaign. Among these, 11 woredas were selected purposively, ensuring representation from the different Regions (Amhara, Oromia and South Ethiopia), and also considering the reported measles case load, immunization service coverage and accessibility. Based on the number of cases reported during the outbreak, 2 health centers/hospitals were selected from each woreda. Within each Health Center’s service catchment area, one or two health posts were included in the assessment depending on the size of the catchment area. Within the villages served by each health post, five households with one or more measles cases aged under 5 years were selected from the outbreak cases line-list at the health facility and/or woreda level, with considerations including easy access. Data collectors visited the households and interviewed the parents or adult caregivers of the identified measles case.

The RCA exercise involved: Interview of the immunisation program focal points and PHEM officers in the 11 selected woredas, interview of the immunisation program focal points and PHEM officers/focals in the selected 24 Health facilities (HC and hospitals), interview of the health extension workers in the selected 27 HPs regarding immunisation and surveillance performance for the 2016 Ethiopian calendar (12 September 2023 - 11 September 2024 European Calendar), review of administrative coverage data from woreda level covering the year 2016 of the Ethiopian calendar (12 September 2023 - 11 September 2024 European Calendar), review of the case-based surveillance data reported from the woreda to the national levels for the year 2024, interview of parents/caregivers of selected children who had been confirmed measles cases during the outbreak (118 households) (Table 1).

**Table 1:** list of selected woredas and health facilities and the number of reported measles cases, 2024

Region	Woreda	Total population (2024)	# of reported measles cases (2024)	# of measles deaths in 2024 (CFR%)	Health facilities and households included in the root cause analysis exercise		
					# of Health Centers/ Hospitals	# of Health Posts	# of households with measles cases
**Amhara**	Argoba	50,082	17	1 (5.9%)	2	3	12
	Habru	273,199	21	0 (0%)	3	3	11
	Lay Armacheho	156,232	71	0 (0%)	2	4	15
	Tsagbji	59,499	168	0 (0%)	1	2	10
**Oromia**	Ambo town	119,751	1063	2 (0.2%)	3	2	10
	Mada walabu	104,103	137	0 (0%)	2	3	10
	Robe Town	93,000	602	4 (0.7%)	3	2	10
	Shala	240,939	946	6 (0.6%)	2	2	10
	Tiro Afeta	181,835	951	1 (0.1%)	2	2	10
**South Ethiopia**	South Ari	105,264	650	10 (1.5%)	2	2	10
	Uba Debre Tsehay	99,936	38	0 (0%)	2	2	10
**Grand Total**			**4664**	**24 (0.5%)**	**24**	**27**	**118**

Data collection and analysis: National program staff provided training to regional and zonal PHEM officers who conducted the data collection. Semi-structured questionnaires designed in the Open Data Kit (ODK) mobile data collection platform were used. The data collection was conducted from October to November 2024. Data collectors uploaded data to the central server on a daily basis. The national team reviewed the data daily and provided feedback on any data quality issues identified. The data analysis was done using MS EXCEL.

## Results

All of the woredas are predominantly rural except Ambo and Robe Towns in Oromia Region. The average population size in the study woredas was 126,914 and ranged from 50,082 people in Argoba Woreda to 273,199 people in Habru Woreda. In 2024, a total of 4664 confirmed measles cases and 24 deaths (CFR-0.5%) were reported to the national level from the 11 woredas. The highest number of measles cases were reported from Ambo Town (1063 cases) followed by Tiro Afeta Woreda (951 cases) and Shala Woreda (946 cases), all in Oromia Region (Table 1).

**Measles surveillance and outbreak investigation:** the assessment looked at the availability of the outbreak line-list of measles cases for the period 12 September 2023 - 11 September 2024 (equivalent to the year 2016 in the Ethiopian Calendar). Two of the 11 woredas didn’t keep line lists of reported measles cases at the woreda health office level- Tiro Afeta Woreda and Ambo Town (both from Oromia region), with the reason given being a lack of awareness. On the other hand, 17 of the 24 health facilities kept the line list of reported measles cases, with the exceptions being five health facilities in Oromia Region and 2 from South Ethiopia Region. Only 5 out of 11 woredas reported having investigated suspected measles cases within 48 hours of notification of cases during the 2023/2024 outbreaks, while the remaining six woredas reported late or no investigation at all. The reasons for delayed or lack of outbreak investigation at the woreda level include lack of operational funding, lack of means of transport for health workers to reach affected areas, overlapping public health activities, and security challenges (conflict or political instability that restricted health workers’ movement).

Vaccination coverage: the administrative report from the 11 assessment woredas for the 2016 Ethiopian Fiscal Year showed that Lay Armachiho woreda of the Amhara region had less than 80% MCV-1 coverage for the last five years (from 2020 to 2024). Some woredas had >100% MCV1 coverage (eg, Mada Walabu, Tiro Afeta, and Shala Woredas of Oromia) throughout the five years. Regarding MCV2 coverage, only 1 woreda (Mada Walabu Woreda of Oromia Region) reported more than 95% administrative coverage in each of the last five years, while most of the other woredas had less than 80% MCV2 coverage in 2020 and 2021 (Table 2, Table 3).

**Table 2:** measles first dose (MCV1) vaccination administrative coverage in the surveyed woredas. 2019/2020 - 2023/2024, Ethiopia, root cause analysis exercise, 2024

Region	Zone	Woreda	2019/2020	2020/2021	2021/2022	2022/2023	2023/2024
Amhara	Central Gondar	Lay Armacheho	69%	54%	57%	72%	63%
	North Wollo	Habru	101%	47%	111%	106%	101%
	South Wollo	Argoba	97%	83%	132%	121%	144%
	Waghimera	Tsagbji	93%	27%	0%	40%	125%
Oromia	E. Borena Zone	Mada walabu	184%	174%	152%	128%	126%
	Jimma Zone	Tiro Afeta	107%	108%	108%	127%	115%
	Robe Town	Robe Town	100%	118%	46%	187%	104%
	West Arsi Zone	Shala	106%	108%	99%	109%	109%
	W/Shewa Zone	Ambo	110%	91%	69%	79%	101%
South Ethiopia	Ari	South Ari	91%	95%	96%	96%	104%
	Goffa	Uba Debre Tsehay	94%	111%	87%	84%	91%

**Table 3:** measles second dose (MCV2) vaccination administrative coverage in the surveyed woredas 2019/2020 - 2023/2024, Ethiopia, Root Cause Analysis exercise, 2024

Region	Zone	Woreda	2019/2020	2020/2021	2021/2022	2022/2023	2023/2024
Amhara	Central Gondar	Lay Armacheho	59%	46%	49%	70%	63%
	North Wollo	Habru	56%	32%	88%	86%	89%
	South Wollo	Argoba	64%	55%	112%	70%	133%
	Waghimera	Tsagbji	74%	21%	0%	26%	114%
Oromia	E. Borena Zone	Mada walabu	136%	144%	128%	105%	102%
	Jimma Zone	Tiro Afeta	71%	65%	75%	97%	94%
	Robe Town	Robe Town	35%	73%	33%	91%	92%
	West Arsi Zone	Shala	113%	69%	66%	89%	91%
	W. Shewa Zone	Ambo	87%	70%	82%	75%	107%
South Ethiopia	Ari	South Ari	68%	88%	84%	85%	100%
	Goffa	Uba Debre Tsehay	89%	82%	72%	77%	87%

Vaccination status of surveyed children: the assessment of vaccination status of the selected 108 children who had measles during the outbreak was done by checking the vaccination cards and/or by verbal recall from the caregivers. The assessment showed that 67 (56%) had received at least one dose of measles vaccine before their illness. Among the children from South Ethiopia Region, 17 (85%) gave history of having received at least one dose of measles vaccination before the illness, while in Amhara region, only 22 (45%) had received one or more measles vaccine doses.

### Factors related to Vaccination Service Providers

**Health facility staffing and immunisation planning:** nearly all (90%) of the woredas have a designated EPI focal person or officer. Only 16 of 24 HFs (59%) and from 27 health posts only 18 (67%) of health posts have an EPI micro plan for 2023/2024 (2016 Ethiopian calendar). None of the health facilities from Udot Woreda, Argoba Woreda, and South Ari Woreda have micro plans. None of the health posts from South Ari and Uba Debre Tsehay woredas and two-thirds of health centers and health posts from Habiru Woreda have micro-plans. The reasons for the lack of micro-plans were not captured during the analysis.

**Health worker capacity:** nineteen (79%) of the 24 EPI focal persons in the assessed health facilities have received the standard “Immunisation in Practice” training. On the other hand, none of the woreda PHEM focal persons had been trained on vaccine-preventable diseases (VPD) or on measles surveillance and response. In addition, none of the PHEM focal persons in the visited health facilities in Amhara Region had received training on measles surveillance and outbreak response.

**Immunization service delivery:** for the period of the 2016 Ethiopian calendar, out of 24 facilities assessed, 17 (71%) provide EPI services daily, while 3 health facilities (13%) do so twice a week (Table 4). More than half (55%) of the assessed 11 woredas do not have any facility that offers daily vaccination services, while the remaining 6 woredas (45%) have at least one facility that provides daily vaccination services. The main reasons cited for not providing daily vaccination services include staff shortage (36%), concerns of vaccine wastage (36%), and lack of operational funding (18%). Twelve (44%) out of the 27 health posts and one-third (8 of 24) of the health centers do not give measles vaccines in every vaccination session (Table 4). Fourteen of 24 health facilities (58%) reported that they open measles vials when five or more children are available at the vaccination session, while 4 (17%) health facilities require 7 or more children. None of the health facilities open 10-dose measles vials with less than 5 clients at the vaccination site (Table 5).

**Table 4:** number of health facilities by woreda, frequency of routine immunisation service delivery and status of MCV administration in every session: Ethiopia, Root Cause Analysis exercise (2016 Ethiopian calendar (2023/2024))

Region	Woreda	Frequency of routine immunization service delivery					# health facilities providing measles vaccination in every immunisation session	
		Daily	Twice weekly	Weekly	Monthly	Other	Yes	No
Amhara	**Argoba**	1		1			2	0
	**Habru**	2	1				2	0
	**Lay Armacheho**	2					2	0
	**Tsagbji**	1				1	1	1
Oromia	**Ambo**	2					2	0
	**Mada Walabu**	1					1	0
	**Robe Town**	3					3	0
	**Shala**	1	1				2	0
	**Tiro Afeta**	2		1			1	2
South Ethiopia	**South Ari**	1	1				1	1
	**Uba Debre Tsehay**	1			1		2	0
Total		**17**	**3**	**2**	**1**	**1**	**16**	**8**

**Table 5:** number of children required to open measles vaccine vials in the 24 health facilities. Ethiopia, Root Cause Analysis exercise, 2024

Region	Woreda	Minimum number of children required to open a 10-dose measles vaccine vial			
		5 children	6 children	7 children	8 children
Amhara	Argoba	1			1
	Habru	3			
	Lay Armacheho	1	1		
	Tsagbji	1	1		
Oromia	Ambo	2			
	Mada Walabu	1			
	Robe Town	2		1	
	Shala	1	1		
	Tiro Afeta	1	1		1
South Ethiopia	South Ari	1			1
	Uba Debre Tsehay		2		
Total		14	6	1	3

Cancellations of vaccination sessions were reported from 24 health centers and 27 health posts in 4 woredas during the 2016 Ethiopian Calendar (12 September 2023 - 11 September 2024). A total of 899 vaccination sessions were canceled within this period, with the highest number of cancellations reported from Shala Woreda, Oromia region (Table 6). The study did not document data with regards to the overall number of planned sessions in these woredas. The most common reasons for the cancellation of vaccination sessions during this time period included staff shortage and vaccine stockout, followed by lack of means of transportation for field trips, electric power interruption, and concerns over vaccine wastage as reported by EPI focal persons at health facilities and health post level.

**Table 6:** number of vaccination sessions cancelled in the selected Health Facilities, shown by woreda (2016 Ethiopian Calendar (12 Sept 2023-11 Sept 2024))

Region	Woreda	Immunization session cancellation			
		Fixed health facility sessions	Outreach sessions	mobile sessions	Total
Amhara	**Argoba**	6	0	0	6
	**Habru**	0	0	0	0
	**Lay Armacheho**	0	0	0	0
	**Tsagbji**	20	7	6	33
Oromia	**Ambo**	0	0	0	0
	**Mada Walabu**	0	0	0	0
	**Robe Town**	1	0	0	1
	**Shala**	408	396	0	804
	**Tiro Afeta**	0	0	0	0
S**outh** Ethiopia	**South Ari**	20	35	0	55
	**Uba Debre Tsehay**	0	0	0	0
Total		**455**	**438**	**6**	**899**

**Vaccine and cold chain logistics:** five out of 24 (21%) health facilities have reported measles vaccine stockouts at least once in the fiscal year. The duration of vaccine stockout varies between 3 days in Senbete Shalla Health Center (Shala Woreda of Oromia Region), 30 days in Medina Health Center (Argoba Woreda of Amhara Region) and 3 months in Gobera Health Center (Argoba Woreda of Amhara Region). Upon assessing the functionality of cold chain equipment in health facilities, four health facilities have reported failure of vaccine refrigerators (during 2016 Ethiopian Calendar). These lasted 2 days, 20 days, 2 months and 3 months respectively, with Awara Health Center in Shala Woreda having the longest period of non-functioning cold chain equipment.

### Factors related to vaccination service clients

The interview of the caregivers/mothers of selected children who had measles during the outbreak included a review of vaccination status (by card/history), and inquiring about reasons for non-vaccination. Accordingly, the long distance to the health facilities was mentioned in 17% of responses, while other reasons include competing engagements for the parents/caregivers, lack of knowledge about the vaccine. Nine caregivers mentioned the absence of health workers or service cancellation during the health facility vaccination visits as a reason for non-vaccination (Table 7).

**Table 7:** parents/caregivers’ reasons for not having vaccinating eligible children who later contracted measles, Ethiopia (N=51), Root Cause Analysis exercise, 2024

Reason for non-vaccination	Frequency	Percentage
Long distance to the Health Facility	12	17%
Parent/ caregiver busy with other priorities	10	14%
Lack of knowledge about the importance of vaccines	14	20%
Child age was not eligible for measles vaccine	10	14%
Health workers / vaccination service not available at the time of the visit	9	13%
Child not in the area at the age for vaccination	5	7%
Parent/ caregiver does not believe in vaccine effectiveness	4	6%
Fear of vaccine side effects, fear of contracting COVID-19	4	6%
The child was sick on the vaccination date	2	3%
Don’t know the reason	1	1%
Total	**71**	**100%**

## Discussion

Root cause analysis is a practical approach to problem solving that involves using any of the different quality control tools to identify and address problems within any process. The “5-Whys” technique is commonly employed when trying to get to the underlying causes of problems. Using the 5-Whys method can lead from the obvious proximate cause to the ultimate underlying cause [13]. This study used a combination of approaches including exploring the various inputs and dimensions in the process of vaccination service delivery, along with a little bit of the 5-Why’s approach. In the review of the administrative vaccination coverage, it was noted that most of these woredas have reported coverage more than 100% for the past few years, indicative of major challenges with the accuracy of the population denominator figures utilized for the catchment areas, and/ or service delivery statistics that captures more children than the catchment population. The last census in the country was in 2007 and so it is likely that population projection figures may differ from the current reality by a wide margin, especially in areas where large population movement occurs for different reasons. On the other hand, Lay Armacheho and Tsagbji Woredas had very low MCV1 and MCV2 administrative coverage reported in the years 2020/2021 to 2022/2023, a period corresponding to the armed conflict in Northern Ethiopia [14].

On the other hand, we note that only 67 (56%) of the identified measles cases in these woredas had received at least one dose of measles vaccine before their illness. This figure varies across the different woredas, and it may be confounded by recall bias (since not all clients could present vaccination cards at the time of the assessment). The study did not look at the timing of receipt of the first dose measles vaccine. However, a significant proportion of children were not protected by vaccine doses. In 2015, a study analyzing age of vaccination as reported in the demographic health survey (DHS) data identified that Ethiopia had >25% invalid measles first dose administration, i.e., doses administered before the recommended age of eligibility and likely to have suboptimal protection [15].

Even though only a small number of woredas and health facilities were covered in this assessment, we note that the measles outbreak in the target woredas was due to “failure to vaccinate” as a primary cause. And according to the findings, the main contributing factors for this failure to vaccinate include various combinations of service delivery gaps: shortage of immunisation service delivery staff, lack of routine immunization micro-plan in some health facilities and woredas, exaggerated administrative vaccination coverage figures, vaccine stockouts, cold chain equipment breakdown, infrequently held vaccination sessions, failure to provide measles vaccination in all sessions, waiting for at least 5 clients before opening measles vaccine vials, and cancelled vaccination sessions. These are compounded by the following root causes: suboptimal level of training of EPI focal points and PHEM focal persons, use of inaccurate denominators for coverage monitoring, lack of funding and means of transportation for outreach vaccination and outbreak investigation activities, fear of vaccine wastage and multiple competing priorities. As seen in the results of the RCA, these multiple root causes are present in varying degrees in the different health facilities and woredas.

Service delivery gaps like vaccine stockouts and cancelled vaccination sessions all contribute to missed opportunities for vaccination, which is a major contributor to immunization coverage gaps. The problem of cancelled vaccination sessions was identified as a weakness in the 2021-2025 comprehensive Multi-Year Plan for Immunisation (cMYP) in Ethiopia [1]. The challenge of missed opportunities for vaccination has been well documented in many studies across Africa. A study in Jimma in Oromia Region of Ethiopia identified 39.8% prevalence of missed opportunities for vaccination among children aged less than 2 years of age who came to health facilities to get different health services [16]. Another qualitative study from Ethiopia documented frequent cancellation of outreach vaccination services because of lack of funding and means of transportation for health workers, similar to what was identified in this root cause analysis exercise [17]. Similarly, a qualitative study from Kenya identified vaccine stockouts, geographic inaccessibility, restricted clinic hours, and limiting the provision of certain vaccines only to specific days of the week as reasons for client dissatisfaction and missed opportunities for vaccination [18]. In Mogadishu, Somalia, 48% of missed opportunities for vaccination were found to be due to health facility-related factors including stock out, not opening vaccine vial for one child, unclear working hours [19]. The national EPI implementation guideline in Ethiopia states that vaccination services should be provided every day and that any health workers should open measles and BCG vaccine vials even if one eligible child presents for measles or BCG vaccination [20]. However, the RCA study confirmed that none of the 24 health facilities open 10-dose vials when there are 5 or less children at the vaccination site, and many health facilities do not provide daily vaccination services. The use of 5-dose vials has been shown to improve the readiness of health workers to open vials with few children at the vaccination post and contributes to improving vaccine coverage and reducing wastage [21,22]. As of April 2025, Ethiopia is preparing to shift to using 5-dose measles vaccine vials for routine immunization services starting in late 2025. However, this shift should go along with encouraging, training and supporting health workers to open these 5-dose vaccine vials readily, to use MCV in all vaccination sessions, and to provide vaccination services daily. The change of vial formulation by itself will not improve coverage without any change in health worker behavior.

Distance to health facilities is an important client-based factor contributing to “failure to vaccinate”. Okwaraji *et al*. found that travel time to health facilities is significantly associated with childhood vaccination coverage in rural Ethiopia, with children living more than an hour away from a health post being significantly less likely to receive BCG, third dose of Pentavalent vaccine and measles vaccines [23]. Metcalf *et al*. also showed how travel time and living further away from towns and cities negatively affects vaccination coverage [24]. Another client-based factor is that caregivers often have other household, farming and / or childcare responsibilities, which makes it essential for them to prioritize when a vaccination schedule is due. This was documented in a study in Malawi [25]. These findings demonstrate the importance of scheduling vaccination sessions and of setting up outreach or mobile vaccination sites in a way that can address the barriers for access and utilization by catchment populations. The reduction of zero-dose children and the attainment of measles elimination can only be attained by organizing vaccination services that can reach previously unreached children.

## Conclusion

As noted in this study, there are common cross-cutting and systemic factors in various woredas, that contributed to the measles outbreaks. However, there are also specific and contextual factors related to measles vaccination which differ from one health facility to another and between woredas, and which need to be linked to tailored efforts to strengthen routine immunisation services within the service catchment areas. We recommend that RCA should be implemented systematically as part of the immediate outbreak investigation and response effort, and the findings in each area should be used to address the program gaps within the specific context [12].

The current root cause analysis exercise has some limitations. The exercise did not look extensively at vaccine cold chain management and vaccine handling practices, which could have identified any factors related to vaccine failure. While documenting vaccine stockouts, the assessment did not explore the underlying reasons behind the vaccine stockout. In addition, in future RCA exercises, it will be important to explore other program elements like the frequency of supportive supervision from the higher levels, the presence of defaulter tracking systems, cold chain functionality, and temperature monitoring systems. In addition, the study woredas, health facilities, and households were all selected based on convenience sampling, and so do not necessarily represent the situation in other outbreak woredas or their respective Regions.

### What is known about this topic

- Measles outbreaks commonly occur because of immunity gaps created when eligible children do not receive vaccines;
- Missed opportunities for vaccination continue to be important factors for low vaccination coverage;
- Root cause analysis exercises are well-established tools for problem solving.

### What this study adds

- Root cause analysis can help identify the underlying contextual problems related to gaps in vaccination within a specific district or a health facility context;
- Infrequent or cancelled vaccination sessions, failure to readily open 10-dose vaccine vials to vaccinate eligible children, and vaccine stock-outs create missed opportunities and are major contributory root causes for measles outbreaks;
- Client-based factors contribute to poor vaccine uptake, and these need to be considered in organizing vaccination sessions and vaccination sites.
